# Oxygen kinetics during 6-minute walk tests in patients with cardiovascular and pulmonary disease

**DOI:** 10.1186/1471-2466-14-167

**Published:** 2014-10-29

**Authors:** Lukas Kern, Sophie Condrau, Florent Baty, Jan Wiegand, Arno JR van Gestel, Andrea Azzola, Michael Tamm, Martin Brutsche

**Affiliations:** Division of Pulmonary Medicine, Cantonal Hospital Zug, Zug, Switzerland; Division of Internal Medicine, Regional Hospital Biel, Biel, Switzerland; Division of Pulmonary Medicine, Hospital St. Gallen, CH-9002 St. Gallen, Switzerland; Division of Critical Care Medicine, University Hospital Bern, Bern, Switzerland; Department of Health, Zurich University of Applied Sciences, Winterthur, Switzerland; Division of Pulmonary Medicine, Regional Hospital Lugano, Lugano, Switzerland; Division of Pulmonary Medicine, University Hospital Basel, Basel, Switzerland

**Keywords:** Oxygen uptake kinetics, 6-minute walk test, Mobile cardiopulmonary monitoring, Submaximal exercise, Cardiopulmonary exercise testing

## Abstract

**Background:**

The 6-Minute Walk Test (6MWT) is representative of daily-life activities and reflects the functional capacity of patients. The change of oxygen uptake (VO_2_) in the initial phase of low-intensity exercise (VO_2_ kinetics) can be used to assess submaximal exercise performance of patients.

The objective of the following study was to analyse VO_2_ kinetics in patients with different pulmonary and cardiovascular diseases. In addition, we investigated the extent to which VO_2_ kinetics at the onset of the 6MWT were associated with exercise capacity, morbidity and mortality.

**Methods:**

VO_2_ kinetics of 204 patients and 16 healthy controls were obtained using mobile telemetric cardiopulmonary monitoring during a 6MWT. A new mean response time (MRT) index (wMRT) was developed to quantify VO_2_ kinetics by correcting MRT for work rate. The differences in wMRT between disease categories as well as the association between wMRT and patients’ exercise capacity and outcome - time to hospitalization/death- were tested.

**Results:**

The assessment of a robust wMRT was feasible in 86% (244/284) patients. wMRT was increased in patients compared to healthy controls (p <0.001). wMRT was largest in patients with pulmonary arterial hypertension (PAH). There were significant associations between wMRT and exercise capacity in all patients. High wMRT was found to be associated with a high rate of death and re-hospitalization in patients with CHF (p = 0.024). In patients with pulmonary diseases and pulmonary hypertension wMRT was not associated with outcome (p = 0.952).

**Conclusions:**

Submaximal exercise performance of patients is reduced. O_2_ kinetics at the onset of exercise are associated with exercise capacity in all patients. wMRT was found to be an important prognostic factor in patients with congestive heart failure (CHF), but not with pulmonary diseases.

## Background

Cardiopulmonary exercise testing (CPET) has been classically performed on a cycle-ergometer or motorised treadmill using a rapid ramped incremental protocol to the limit of tolerance [1]. Since most activities of daily living are performed in a non-incremental fashion and at submaximal level of exertion, the 6-Minute Walk Test (6MWT) may be more representative of daily-life activities and may reflect the functional capacity of patients more accurately [2]. In addition, because most activities of daily living require repetitive submaximal effort, it may be postulated that analysing the cardiopulmonary responses during a transition from rest to submaximal intensity work may provide important information. Therefore, there is a growing interest in submaximal exercise parameters capable of reflecting the functional capacity of patients.

The change of oxygen uptake (VO_2_) during constant work exercise (VO_2_ on-kinetics) is classically subdivided into three functionally distinct phases [3, 4]. A rapid first phase characterized by a short time delay of approximately 20 seconds reflects an increase in pulmonary blood flow. After the first phase, O_2_ uptake gradually increases in an approximately mono-exponential fashion (phase 2) until a steady state level (VO_2SS_) (phase 3) is attained. Phase 2 reflects the ability of the cardiopulmonary system to deliver oxygen and the amount of oxygen that is utilized by skeletal muscles. At the onset of exercise there is a delay in skeletal muscle mitochondrial ATP production due to a limited oxygen supply (oxygen deficit). Until the steady state is attained, anaerobic glycolysis compensates for this short-term oxygen deficit [5, 6].

VO_2_ on-kinetics can be characterized by the time (mean response time: MRT) required for VO_2_ to achieve 63% of the VO_2SS_ in response to physical stress [7, 8]. MRT is usually calculated in a CPET setting using a constant work rate protocol [8, 9]. As the work rate during the 6MWT is mostly determined by the patients’ individual effort, MRT has to be calculated differently. In this study, a new MRT index (wMRT) is proposed to quantify VO_2_ kinetics at the onset of exercise by correcting MRT for work rate during the first phase of the 6MWT.

It has been demonstrated that patients with chronic pulmonary or cardiac disorders exhibit slower VO_2_ on-kinetics [10, 11] when compared to healthy age-matched controls.

More knowledge of the physiological determinants of O_2_ uptake kinetics at the onset of exercise may lead to a better understanding of the pathophysiological mechanisms causing functional impairments in patients. The importance of assessing VO_2_ kinetics in the initial phase of low-intensity exercise may be underlined by that fact that peak VO_2_ during a maximal exercise test is influenced by conditions other than the underlying disease, the patient’s motivation and the criteria used to terminate the test [12]. In addition, it has been demonstrated in several chronic diseases that VO_2_ on-kinetics have a higher prognostic value than peak VO_2_ [13].

No data exist about the clinical utility of this refined method to quantify functional capacity of patients. Therefore, the main purpose of the present study was to analyse VO_2_ kinetics at the onset of exercise in patients with different pulmonary and cardiovascular diseases using mobile telemetric cardiopulmonary monitoring (MOB). Given the vast pathophysiological heterogeneity of these diseases it may be reasonable to presume that different diseases may have different VO_2_ kinetics. Secondarily, we investigated the extent to which VO_2_ kinetics are associated with exercise capacity (VO_2SS_ and 6-Minute Walking Distance, 6MWD). The third aim of the present study was to investigate if VO_2_ kinetics are associated with morbidity and mortality in these patients.

## Methods

### Study design

**Figure 1:**
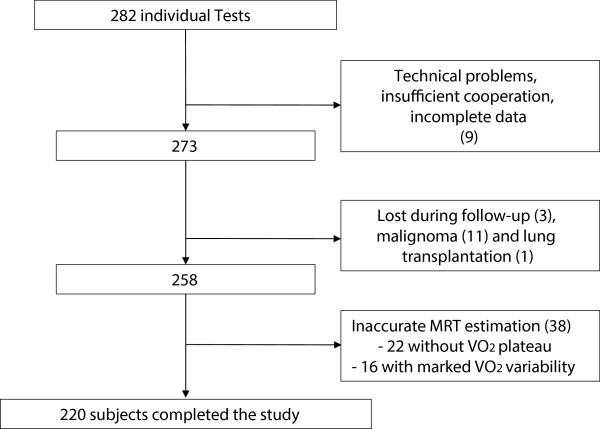
**Study exclusions due to technical issues or loss of follow-up.** Nine (3%) tests were excluded due to significant patient-related or technical flaws. In thirty-eight (15%) tests it was not possible to fit an acceptable oxygen uptake kinetics curve. Two hundred and twenty tests (82%) were included in the final analysis.

Patients referred to the Pulmonary Division of the University Hospital Basel, Switzerland between August 2003 and June 2007 were considered for participation in the study (Figure 1). Healthy volunteers were recruited from among the hospital staff and college students. Exclusion criteria were as follows: need for oxygen supply or resting transcutaneous oxygen saturation (SpO_2_) of <85% while breathing room air, inability to walk, any acute coronary event during the previous month and conditions precluding the use of a face mask (e.g. anatomic anomaly, claustrophobia or panic disorder). The follow-up was performed by interviewing the referring physician and/or by chart review.

All patients gave informed consent. Our local institutional review board (Ethikkommission beider Basel (EKBB)) approved the study. Parts of the study population data had also been used in another study evaluating the feasibility and safety and clinical usefulness of a MOB-enhanced 6MWT [14].

### Six-minute walk test

The 6MWT was performed according to the guidelines published by the American Thoracic Society (ATS) [2] with a standardized encouragement in a 30 m corridor. All tests were conducted by the same experienced technician. Before starting the 6MWT, patients rested seated for three to five minutes until stable VO_2_ values were recorded (VO_2_rest). Spirometry was performed prior to the test. The 6MWT was followed by a recovery phase, where the patient rested on a chair. Total walking distance, Borg's dyspnoea score and the subjective limiting factor were recorded.

### Six-minute walk test with MOB device

We used the Oxycon Mobile® (Viasys Healthcare, USA) portable, wireless cardiopulmonary exercise testing device to measure breath-by-breath oxygen exchange kinetics. Pulse rate was determined using an ECG-triggered belt (Polar® Electro OY T-61). SpO_2_ was measured using a finger clip. VO_2_ and carbon dioxide output (VCO_2_), tidal volumes and breathing frequency were assessed using a facemask (dead space <70 ml) with a flow sensor and a gas analyser. The patient carried data storage and transfer units by using a dedicated harness. Wireless transfer of breath-by-breath data to a laptop computer allowed real-time monitoring. The additional weight (950 g) of the equipment has no effect on walking distance [14]. The exact 6MWT-MOB procedure has been described in previously published work [14].

ATS criteria for the determination of test intensity were applied [1]: the effort was considered to be maximal if either one or more of the following criteria were fulfilled: (1) maximal heart rate >90% predicted, (2) VO_2_peak >84% of the predicted maximum, and (3) ventilatory reserve <11 liters or <15%. Ventilatory reserve, VO_2_max predicted and maximal predicted heart rate were calculated using standard equations [15, 16]. Spirometry was interpreted according to the ATS/ERS guidelines [17].

### Curve fitting of oxygen uptake

**Figure 2:**
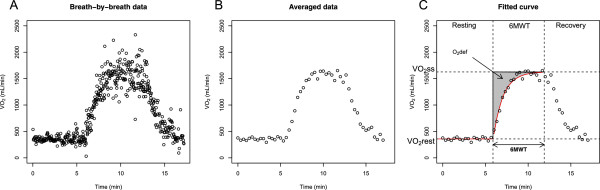
**Pre-processing of oxygen uptake during 6MWT and curve fitting.** Panel **A** shows the raw breath-by-breath data. Panel **B** displays the data after averaging over successive periods of 18 seconds. Panel **C** depicts the curve fitting (red line) during the 6-minute walk test (6MWT), together with the parameter estimates including the lower limit (y1), the upper limit (y2), and the oxygen deficit (O_2_def), from which the mean response time is derived.



Original breath-by-breath data were imported from the MOB device. Raw data were pre-processed by averaging the breath-by-breath measurements over consecutive periods of 20 seconds (Figure 2, panel A and B). Curves were modelled using asymptotic regression models describing the oxygen uptake during the 6MWT.

with y_1_ the lower limit at *t* = 0, y_2_ the upper limit, and the parameter *τ* >0 determining the steepness of the increase as *t* elapses. In physiological terms, this translates into:


with VO_2_rest the oxygen uptake at rest, and VO_2ss_ the oxygen uptake at steady state during exercise.

Notice that the phase I classically characterized by a short time delay was not modelled in the current study. This time delay was undistinguishable from the second exponential phase as often happening in vivo [18]. A gain of stability was obtained by not using an over-parameterized model, which over compensated the potential risk of slight systematic over estimation of *τ*.

The oxygen deficit (O_2_def) is defined as the area between an instantaneous increase of oxygen to the maximum upper limit and the observed asymptotic rise of oxygen (Figure 2, panel C) [19]. Since no time delay is taken into account, the mean response time (MRT) corresponds to the time constant (*τ*) of the exponential function describing the rate of oxygen increase [10]. It represents to the time needed for a 63% increase in oxygen uptake and can also be defined as follows:


Models were fitted on VO_2_ kinetics using nonlinear regression with the standard Gauss-Newton optimization algorithm and assuming error terms which are normally distributed, mutually independent, centred around 0 and of unknown variance. Each individual curve was analysed graphically and the goodness-of-fit was first assessed visually and with the help of the Neill’s lack-of-fit test [20].

### Mean response time reparameterization

In this study, a new MRT index (wMRT) was developed to quantify VO_2_ kinetics at the onset of exercise by correcting MRT for work rate during the first phase of the 6MWT. The difference between O_2_ uptake at rest and during effort (VO_2_ss - VO_2_rest) was used as a proxy for work rate during the first phase of the 6MWT. MRT was then reparameterized as follows:


MRT reparameterization using other variables such as walking distance (WD) was not possible, probably because of the fact that WD reflects total exercise capacity rather than the initial phase of a submaximal exercise test and is also influenced by muscular or skeletal function as well as motivational level.

### Statistical data analysis

Descriptive data for continuous variables are expressed as mean, standard deviation and percentages for frequencies. Variables were tested for parametric distribution by applying the Shapiro-Wilk Test (null hypothesis rejection set at p < 0.25). Univariate linear regression was performed to evaluate possible associations between wMRT and measures reflecting exercise capacity (VO_2SS_, 6MWD). A p-value of <0.05 was considered to indicate statistical significance. Differences between continuous variables were tested using linear models (ANOVA F-test). Time-to-event data was modelled using Kaplan-Meier estimators, and hypothesis testing was performed using Cox-proportional hazards regression (Wald test). All the analyses were done using the R statistical software [21], including the extension package *drc* for curve fitting [22] and *nlstools* for the diagnoses of the quality of fit in nonlinear regression [23].

## Results

### Fit of oxygen kinetics and patient characteristics

Two hundred and eighty two individuals were examined. Nine patients had to be excluded for reasons of technical failure, insufficient cooperation or incomplete data. Fifteen patients were excluded due to loss of follow-up, lung transplantation and diagnosis of malignancy. The quality of fit was first assessed visually by two independent investigators in order to detect fits showing obvious lack-of-fit. In thirty-eight patients from all categories except controls, curve fitting did not succeed, leading to exclusion from the analysis. Two distinct patterns of response to exercise were noticed in this group: in twenty-two patients, oxygen uptake did not show a plateau and in sixteen patients, VO_2_ showed a marked variability after reaching a plateau. Variable walking speed and pauses during the 6MWT are the most likely explanations for this observation. The exclusion process is depicted in Figure 1.

The goodness-of-fit of each of the remaining 220 curves was further assessed using Neill’s lack-of-fit test for nonlinear regression. This test showed no evidence of lack-of-fit for all curves but four, where the Neill’s test provided significant results (adjusted p-values <0.05). These four curves were nevertheless kept in the final analysis as their lack-of-fit was not considered major, and was not significantly impacting the estimate of the model parameters.

Two hundred and four patients and sixteen healthy controls were included in the final analysis. Patient characteristics are reported in Table 1. One hundred and twenty eight patients (58%) reached criteria for maximal effort according to ATS guidelines [1]. In the current study the mean RER was 0.86 (0.14) suggesting that only a few patients reached an anaerobic threshold. Patients were categorized according to their underlying diseases: thirty nine patients suffered from restrictive lung disease, eighty-four from COPD, fifty-four from pulmonary arterial hypertension (PAH) and twenty-seven from congestive heart failure (CHF).

**Table 1:** **Characteristics of participants**

	Healthy	COPD	Restrictive	PAH	CHF
n	16	84	39	54	27
Sex (females)	8	40	21	36	12
Age (years)	37 ± 12	62 ± 14	63 ± 11	61 ± 14	70 ± 9
BMI (kg/m^2^)	24 ± 4	26 ± 6	30 ± 7	28 ± 6	31 ± 8
FEV_1_(l/s)	3.9 ± 0.9	1.3 ± 0.5	1.8 ± 0.6	1.9 ± 0.7 (n = 53)	2.4 ± 0.8
FVC (l)	5.0 ± 1.3 (n = 15)	2.6 ± 0.8	2.3 ± 0.8	2.8 ± 1.0 (n = 53)	3.3 ± 1.2
FEV_1_/FVC	0.8 ± 0.1 (n = 15)	0.5 ± 0.1 (n = 82)	0.8 ± 0.1	0.7 ± 0.2 (n = 53)	0.7 ± 0.1
VO_2SS_ (ml/min)	2376 ± 630	1031 ± 264	1144 ± 299	961 ± 263	1184 ± 342
VO_2MAX_ (ml/min)	2377 ± 630	1012 ± 272	1156 ± 289	945 ± 261	1191 ± 331
VO_2MAX_ (% predicted)	96 ± 15	60 ± 17	66 ± 19	58 ± 18	66 ± 14
HRmax (%predicted)	86 ± 10	69 ± 13	71 ± 12	73 ± 16	70 ± 13
Walking distance (m)	720 ± 83	357 ± 110	381 ± 111	339 ± 120	341 ± 91
Borg	4.1 ± 2.4	4.8 ± 2.4 (n = 80)	4.7 ± 2.1	4.9 ± 2.6	4.5 ± 2.5
Follow-up time (months)	38 ± 6	16 ± 13	21 ± 16	16 ± 14	14 ± 12
Death (n)	0	4	3	10	0
Hospitalizations during follow-up	0	34	17	27	14
MRT (min)	1.13 ± 0.28	1.06 ± 0.28	1.08 ± 0.32	1.07 ± 0.30	0.92 ± 0.34
wMRT (min^2^/ml)	0.66 × 10^-3^ ± 0.13	2.18 × 10^-3^ ± 1.68	1.72 × 10^-3^ ± 0.81	2.59 × 10^-3^ ± 1.18	1.62 × 10^-3^ ± 0.55

Data are presented as mean (SD). BMI: body mass index; FEV_1_: forced expiratory volume in one second; FVC; forced expiratory vital capacity: FEV_1_/ FVC ratio: forced expiratory volume in 1 sec (FEV_1_) expressed as % of FVC; VO_2SS_ = oxygen uptake at steady state; VO_2MAX_ = maximum oxygen uptake; HRmax: maximum heart rate: MRT: mean response time; wMRT: mean response time corrected for work rate.

The participants are grouped into 5 categories: healthy controls (Healthy); restrictive lung disease (Restrictive); chronic obstructive pulmonary disease (COPD); pulmonary arterial hypertension (PAH); congestive heart failure (CHF).

### Exercise capacity and oxygen kinetics at exercise onset

**Figure 3:**
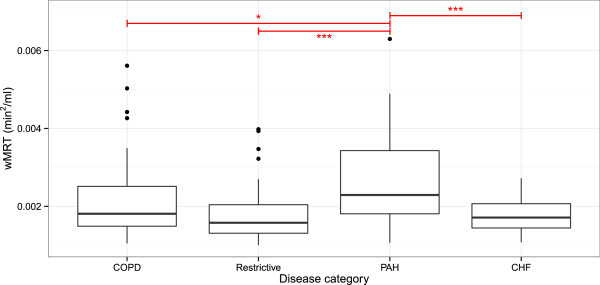
**Mean response time in the various participants’ categories.** The figure shows the box plot distributions of wMRT within the following categories: healthy controls (Healthy); restrictive lung disease (Restrictive); chronic obstructive pulmonary disease (COPD); pulmonary arterial hypertension (PAH); congestive heart failure (CHF). The reparameterized wMRT showed significant differences between patients and healthy controls (p <0.001), the latter showing low wMRT values. Patients suffering from PAH have higher wMRT values than patients with COPD (p = 0.010), restrictive lung disease (p <0.001) and with CHF (p <0.001).

Exercise capacity as assessed by both 6MWD and VO_2SS_ of the patients was significantly reduced compared to that of the healthy controls (Table 1; *p* <0.001). Exercise capacity did not differ between the different patient categories. Figure 3 shows the box plot distributions of wMRT of the patients with restrictive lung disease, COPD, PAH as well as the patients with CHF. The reparameterized wMRT showed significant differences between patients and healthy controls (*p* <0.001), the latter showing low wMRT values. Patients with PAH had significantly higher wMRT values as compared with patients with COPD (*p* = 0.010), patients with restrictive lung disease (*p* <0.001), and patients with CHF (*p* <0.001).

### Association between oxygen kinetics at exercise onset and exercise capacity

**Figure 4:**
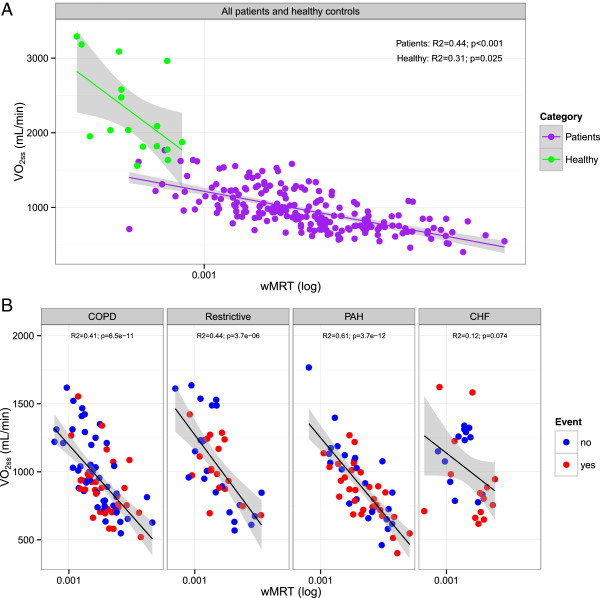
**Scatterplot showing the relationship between wMRT and VO**_**2ss**_ **. A** Scatterplot showing the relationship between wMRT and VO_2SS_ in healthy controls (green dots) and in patients (blue dots). **B** Scatterplot showing the relationship between wMRT and VO_2SS_ in patients with chronic obstructive pulmonary disease (COPD), restrictive lung disease (Restrictive), pulmonary arterial hypertension (PAH) and congestive heart failure (CHF). The red dots in the scatter plot show the patients who suffered an event (death or re-hospitalization) during the follow-up period.

The results of univariate regression analysis are demonstrated in Table 2. The reparameterized wMRT showed a significant association with exercise capacity (VO_2SS_) in all patients (Figure 4A). Figure 4B demonstrates the association between wMRT and VO_2SS_ in the different disease categories. The reparameterized wMRT showed highly significant associations with VO_2SS_ in patients with COPD (*p* <0.001), restrictive lung disease (*p* <0.001) and patients with PAH (*p* <0.001) (Figure 4B). In all patient categories wMRT was significantly associated with 6MWD.

**Table 2:** **Association between wMRT and exercise capacity**

	VO _2SS_(ml/min)			6MWD (m)			MRT (min)		
	β	***p***	R ^2^	β	***p***	R ^2^	β	***p***	R ^2^
COPD	-399.8	<0.001	0.411	199.7	0.007	0.086	0.40	<0.001	0.31
Restrictive	-479.3	<0.001	0.443	503.5	<0.001	0.299	0.35	0.005	0.19
PAH	-425.9	<0.001	0.608	236.5	<0.001	0.208	0.20	0.032	0.09
CHF	-276.7	0.074	0.122	528.7	0.004	0.282	0.31	0.039	0.16

Univariate regression is expressed as β and R^2^. VO_2SS_ = oxygen uptake; 6MWD = 6-minute walk distance; MRT: mean response time; wMRT: mean response time corrected for work rate.

### Morbidity and mortality

**Figure 5:**
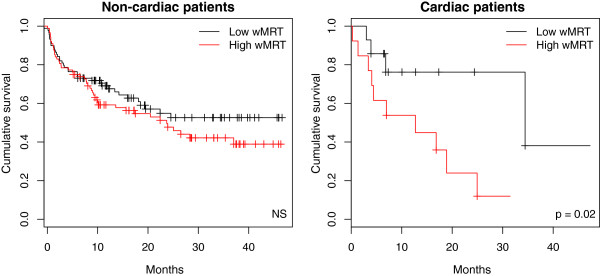
**Reparameterized mean response time and time to death and/or hospitalization during follow-up.** The Kaplan-Meier curves depict the time to death and/or hospitalization in non-cardiac (left panel) and cardiac (right panel) patients, as a function of the reparameterized mean response time (wMRT) dichotomized (based on the median wMRT: non- cardiac patients: 1.85V10^-3^, cardiac patients: 1.59×10^-3^) into low vs. high wMRT categories.

The follow-up was 17 ± 14 months. Seventeen patients (8%) died and 91 (45%) had to be hospitalized during follow-up. Ninety-five patients (47%) reached the combined endpoint of either death or hospitalization. The red dots in Figure 4B show the patients who suffered an event (death or re-hospitalization) during the follow-up period. Figure 5 gives the results of the Kaplan-Meier analysis of the patients with chronic lung disease including pulmonary hypertension and chronic heart failure dichotomized according to the median wMRT (non- cardiac patients: 1.85×10^-3^, cardiac patients: 1.59×10^-3^) into “Low wMRT” and “High wMRT”. No statistical difference was found in patients with chronic lung disease including pulmonary hypertension. In patients with chronic heart disease, a low wMRT was associated with a good outcome, i.e. lower rate death and re-hospitalization (*p* = 0.024). In patients with pulmonary diseases and pulmonary hypertension wMRT was not associated with outcome (*p* = 0.952).

## Discussion

The main purpose of the present study was to analyse VO_2_ kinetics during the 6MWT in a relatively large population of patients with different pulmonary and cardiovascular diseases using mobile telemetric cardiopulmonary monitoring (MOB). A new MRT index (wMRT) was developed to quantify VO_2_ kinetics by correcting MRT for work rate. The relevance of this new parameterization was further confirmed by the reproducibility of the prior results from Schalcher and colleagues [9]. wMRT was lower in all patients’ categories compared to healthy controls. Patients with PAH had a significant higher wMRT value compared to the other patients. We found significant associations between wMRT and exercise capacity in all patients. Furthermore, wMRT was found to be a significant prognostic factor in patients with CHF, but not in patients with lung diseases and PAH.

6MWT is easy to administer, well tolerated, and more reflective of activities of daily living than incremental maximal cardiopulmonary exercise testing (CPET) performed on a cycle-ergometer. In addition, because some individuals may not tolerate individualized maximal incremental exercise testing, analysing VO_2_ kinetics during 6MWT may be a useful alternative to CPET.

There has been controversy about the physiologic responses to the 6MWT in patients and described as both maximal [24, 25] and submaximal [26] sustainable exercise. Interestingly, in this study we found that 128 patients (58%) reached criteria for maximal effort according to the ATS guidelines [1]. Detailed information on factors limiting exercise has been presented in previously published work [14]. Furthermore, although exercise capacity was clearly diminished, no difference was found between the exercise capacity of the patients with different pulmonary and cardiovascular diseases. Despite the vast pathophysiological heterogeneity of these diseases we found that end-exercise oxygen uptake during the 6MWT was not different between patients groups. On the other hand, we found that the O_2_ kinetics at the onset of exercise were clearly different between groups. The results of the present study may, therefore, lead to a better understanding of the pathophysiological mechanisms causing functional impairments in these patients.

The oxygen uptake at the onset of exercise is the product of cardiac output (CO) and the arterial-venous oxygen difference. The relative contribution of Q determinants (i.e., heart rate (HR) and stroke volume (SV)) to oxygen uptake is essential in reducing the O_2_ deficit and corresponding metabolic demand during exercise. The increase in cardiac output at the onset of exercise predominantly depends on the increase in SV and due to an increase in heart rate.

The delay in O_2_ kinetics at the onset of exercise (wMRT) was largest in patients with PAH. A significant difference in wMRT was found between patients with PAH and the rest of the patients. As patients with PAH exhibit impairment in the distensibility and vasodilatory capacity and reduction in the size of the pulmonary vascular bed, the capacity for SV to augment CO is limited [27–30]. In addition, exercise stresses the pulmonary circulation even more causing an abnormal increase in pulmonary artery pressure (PAP) during exercise (exercise-induced pulmonary hypertension) [31–33]. As exercise even further increases PAP during the 6MWT in PAH, this could explain the significant difference in wMRT found between patients with CHF and PAH in the current study.

In patients with pulmonary diseases the delay in O_2_ kinetics at the onset of exercise was associated with both 6MWD and VO_2SS_. Although reduction in exercise capacity in patients with COPD is predominantly related to the combination of increased ventilatory requirements (mainly secondary to increased ventilation/perfusion mismatching) and acute derangements in dynamic ventilatory mechanics, the role of diminished stroke volume on exercise capacity may be underlined by the results of the present study. Borghi-Silva and colleagues [10] have recently demonstrated, that VO_2_ on-kinetics were associated with disease severity in patients with COPD. Hyperinflation may play an important role regarding heart size and heart dysfunction in patients with COPD. Watz and colleagues [11] found that patients with COPD have an impaired left ventricular diastolic filling and an impaired global right ventricular function and that impaired left ventricular diastolic filling was independently associated with a reduced exercise tolerance. These results may eventually aid in the development of therapeutic approaches to improve the exercise capacity in patients with COPD.

The delay in O_2_ kinetics at the onset of exercise in patients with heart failure was associated with 6MWD too. In patients with heart failure, delayed O_2_ on-kinetics may primarily be reduced due to systolic and/ or diastolic left ventricular dysfunction [34, 35]. Controversially, other researchers found that impaired chronotropic and vasodilator reserves limit exercise capacity in patients with heart failure [36, 37].

Besides the changes in SV, a reduced arterial-venous oxygen difference may also play a significant role in the oxygen uptake at the onset of exercise. The latter is dependent on systemic blood flow, the amount of oxygen extraction possible from the systemic capillary blood, and the degree of arterial hypoxemia. Studies evaluating the physiological determinants of O_2_ uptake kinetics showed that abnormal oxidative metabolism at the skeletal muscle level may also contribute to delayed oxygen-uptake kinetics and to the decreased exercise capacity in patients with several chronic diseases [35, 38].

Meyers and colleagues investigated if exercise capacity is predictor of mortality in a total of 6213 healthy subjects and those with cardiovascular disease. In both groups, the peak exercise capacity achieved was a stronger predictor of an increased risk of death than other established risk factors for cardiovascular disease such as hypertension, smoking, and diabetes [39].

In terms of prognosis, our results are in line with the previous findings showing that high MRT values are associated with a bad prognosis in CHF patients [9]. On the other hand, wMRT was not associated with a bad prognosis for patients with lung disease or PAH.

Limiting factors of our study include the fact that MRT could not be calculated in all patients. In about 17% of cases the curve fit failed. Some of these patients were not able to perform the 6MWT with a constant walking speed or without interruptions. This fact may be the reason for the marked variability in VO_2_ in 16 patients in whom curve fitting failed. Those patients whose VO_2_ did not reach a plateau probably increased their walking speed during the 6MWT thus also increasing work rate. In addition they may have been exercising above their anaerobic threshold which permits an upward shift in VO_2_ and an increased demand on central oxygen transport mediated by increased peripheral metabolism. In the current study only 5 of 204 patients (2.2%) reached the level of RER ≥1.10 suggesting that only a few patients reached an anaerobic threshold. In addition, the RER maximal-exercise RER ≥1.10 is commonly used as a criterion to determine whether a “true” VO_2_max has been attained during maximal-effort exercise testing. It should however be stressed, that the use of RER ≥1.10 as a criterion for a valid or “true” VO_2_max in patients with pulmonary diseases has been challenged. Peak exercise performance despite low RER is often seen in patients with a pulmonary limitation to exercise [40].

We did not match age and gender between cases and controls because the purpose of the present study was not to demonstrate exercise intolerance in patients. The current study was in essence “observational”. For this purpose, a cross-sectional design was chosen, using only one single transition per participant. It should be stressed that this may have jeopardized the accuracy and precision of our data.

## Conclusion

In conclusion, no difference was found between the exercise capacity of the patients with different pulmonary and cardiovascular diseases. The delay in O_2_ kinetics at the onset of exercise (wMRT) was largest in patients with PAH. Furthermore, we found significant associations between wMRT and exercise capacity in patients with lung diseases, heart failure and PAH. wMRT was found to be a significant prognostic factor in patients with CHF, but not with pulmonary diseases. The results of the present study may lead to a better understanding of the pathophysiological mechanisms causing functional impairments in these patients. We, therefore, recommend assessing wMRT in future studies investigating exercise capacity, morbidity and mortality in patients with pulmonary diseases and heart failure.
